# Resolving Prokaryotic Taxonomy without rRNA: Longer Oligonucleotide Word Lengths Improve Genome and Metagenome Taxonomic Classification

**DOI:** 10.1371/journal.pone.0067337

**Published:** 2013-07-01

**Authors:** Eric B. Alsop, Jason Raymond

**Affiliations:** School of Earth and Space Exploration, Arizona State University, Tempe, Arizona, United States of America; Université Paris Sud, France

## Abstract

Oligonucleotide signatures, especially tetranucleotide signatures, have been used as method for homology binning by exploiting an organism’s inherent biases towards the use of specific oligonucleotide words. Tetranucleotide signatures have been especially useful in environmental metagenomics samples as many of these samples contain organisms from poorly classified phyla which cannot be easily identified using traditional homology methods, including NCBI BLAST. This study examines oligonucleotide signatures across 1,424 completed genomes from across the tree of life, substantially expanding upon previous work. A comprehensive analysis of mononucleotide through nonanucleotide word lengths suggests that longer word lengths substantially improve the classification of DNA fragments across a range of sizes of relevance to high throughput sequencing. We find that, at present, heptanucleotide signatures represent an optimal balance between prediction accuracy and computational time for resolving taxonomy using both genomic and metagenomic fragments. We directly compare the ability of tetranucleotide and heptanucleotide world lengths (tetranucleotide signatures are the current standard for oligonucleotide word usage analyses) for taxonomic binning of metagenome reads. We present evidence that heptanucleotide word lengths consistently provide more taxonomic resolving power, particularly in distinguishing between closely related organisms that are often present in metagenomic samples. This implies that longer oligonucleotide word lengths should replace tetranucleotide signatures for most analyses. Finally, we show that the application of longer word lengths to metagenomic datasets leads to more accurate taxonomic binning of DNA scaffolds and have the potential to substantially improve taxonomic assignment and assembly of metagenomic data.

## Introduction

Microbes maintain biases in their nucleotide usage that are reflected in their genetic material. These biases were initially noted as the average (G+C) content in prokaryotes, ranging from 17% to 74% [1]. However, biases extend well beyond mononucleotides, to lengths in excess of twenty-five nucleotides in Archaea [2]. These biases are thought to be a result of codon usage patterns due to environmental limitations [3], as well as biases in DNA replication and repair systems [4]. The tetranucleotide biases (signatures) for *Sulfolobus islandicus* and *Escherichia coli* are shown in Figure S1 in comparison to the tetranucleotide signature of a randomly generated 1.6 million base pair DNA sequence, ordered by rank abundance to highlight differences in bin populations between the species and between randomly generated sequences. From these figures it is clear that nature diverges from a uniform distribution of tetramer words and that this divergence varies greatly among the different domains of life.

As oligonucleotide signatures are generally conserved across an organism’s entire genome, they have become a powerful tool for inter-genome comparisons [5]–[16] and as a very useful method for taxonomy-based binning of DNA from environmental metagenomics samples [17]–[20]. This work is absolutely essential to resolving the taxonomic make-up of natural environments, as the DNA/RNA fragments obtained via metagenomics are usually stripped of taxonomically informative genes such as rRNA. Even in metagenomic studies where rRNA libraries are available, connecting an rRNA sequence in one dataset to a metagenomic read in another dataset is non-trivial; rRNA is notably biased in complex communities, over-representing some community members that are easily amplified, and under-representing (or even completely missing) community members whose rRNA is poorly amplified [21]. Much work has been done to develop algorithms for clustering metagenomic data based on statistical correlations of oligonucleotide usage patterns, including self organizing maps [22]–[24] and principal component analysis [25]–[27]. The enormous diversity found in natural communities and the short lengths of metagenome sequencing reads both act to prohibit assembly of metagenomic data into complete genomes. As a result, alternative methods for classifying the organisms in environmental genomics samples have been under rapid development [28]–[31] .

Despite evidence that oligonucleotide signatures of up to eight words in lengths may be useful for clustering [7], [8], [27] most work has concentrated on word lengths of two or four (dinucleotide and tetranucleotide), often without clear rational for not analyzing longer word lengths. Additionally, while a comprehensive analysis was completed to verify the usefulness of tetranucleotide signatures for comparative studies [32], there are no large-scale comparative studies validating tetranucleotide signatures as the optimal oligonucleotide word length for classifying genomes and metagenomes. Furthermore, the recent, dramatic expansion in the availability of sequenced genomes from across the tree of life compels a more comprehensive analysis, undertaken herein, of oligonucleotide biases across a range of word lengths and including all prokaryotic genomes available via NCBI’s publicly available repository.

In this study we have expanded previous oligonucleotide studies to include 1,424 sequenced microbes, including 1,315 bacteria and 109 archaea, analyzing oligonucleotide usage biases from mononucleotides through nonanucleotides. We also examined the extent to which these biases are preserved in varying sized fragments of entire genomes, so as to replicate the smaller fragment sizes associated with metagenome/environmental sequencing and assembly. Based on our findings, we argue that longer word lengths demonstrate the most potential for phylogenetic differentiation and the ability to classify microbes with an accuracy nearing 16S rRNA. These findings underscore the potential applicability of these techniques to metagenomic data sets where sequencing coverage permits assembly of scaffolds of 10 kb or larger. While tetranucleotide signatures are still useful for homology comparisons and are computationally facile to calculate, we argue that longer signatures are well within the range of modern computers and permit more accurate classification of genomes and scaffolds. We identify a tradeoff above word lengths of seven nucleotides where the diminishing increase in taxonomic resolution is not justified by the concomitant exponential increase in calculation time and computational resources required (at least given the present generation of computational facilities). As a result, we recommend the use of longer word lengths with heptanucleotide signatures as the optimal compromise between resolution and computational requirements for future work using oligonucleotide signatures.

## Results and Discussion

**Figure 1:**
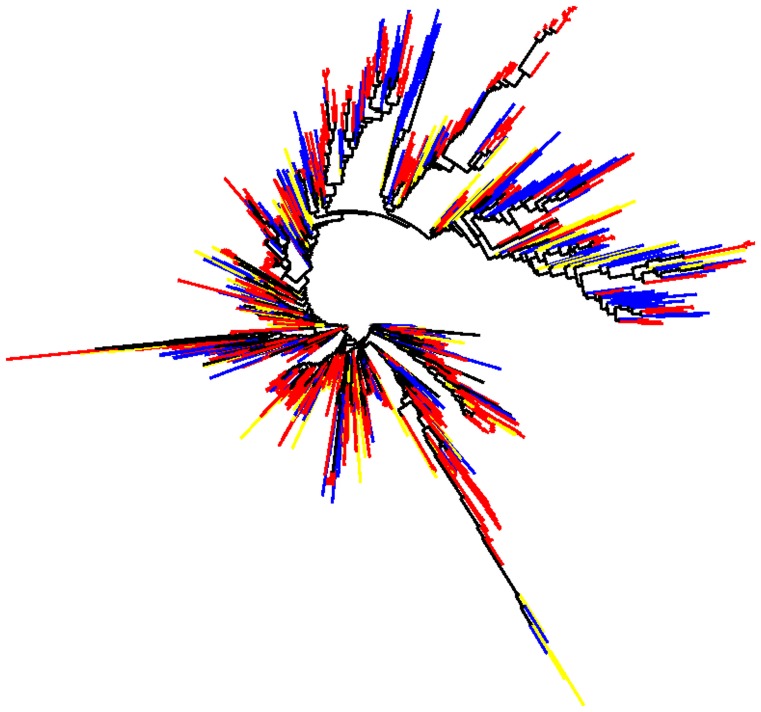
Heptanucleotide Signature Based Cladogram. Cladogram derived from heptanucleotide signatures using Euclidean distances between 1,424 sequenced microbes. Terminal branches are color-coded to depict nearest neighbor taxonomic relationships as: strong relationships (same species or same genus) in red, good relationships (phylum or better) in blue, same domain in yellow and different domain in black. This figure shows that heptanucleotide signatures are conserved amongst phylogenetically similar organisms across the tree of life. The tendency for phylogenetically similar organisms to maintain similar oligonucleotide biases is the basis oligonucleotide-based clustering techniques.

Using oligonucleotide-based Euclidean distance matrices (see Methods), we constructed cladograms to visually represent the clustering ability of various oligonucleotide word lengths. Figure 1 contains a cladogram representing the relationships derived from heptanucleotide signatures (cladograms representing di- through nona- oligonucleotide signatures are included in Figure S2). The terminal branches of all cladograms are color coded based on taxonomy: those with a nearest neighbor from the same genus or species are red (strong relationships), those with nearest neighbors at phylum or better are blue (good relationships), those with nearest neighbors of the same domain are yellow and those with nearest neighbors from different domains are black. It is important to note that in many cases (particularly in the Archaeal domain) a same genus or species nearest neighbor is not possible due to limited availability of sequenced organisms within some phyla. These cladograms demonstrate the power of grouping taxonomically similar microbes based solely on their oligonucleotide signatures: the majority of terminal leaves are colored either red or blue, with many of those colored red (Numerically in Table S1). Comparisons across all oligonucleotide signature lengths demonstrate their effectiveness placing organisms from the same taxonomic groups together, with a general trend towards shorter branch lengths at longer word lengths. Additionally, these cladograms show that oligonucleotide signatures are conserved between closely related microbes across the tree of life. The conservation of signatures among closely related organisms is the key to using oligonucleotide signatures as a method for determining taxonomy, and it is noteworthy that genomes across the tree of life show distinct, evolutionarily conserved trends in their oligonucleotide biases.

**Figure 2:**
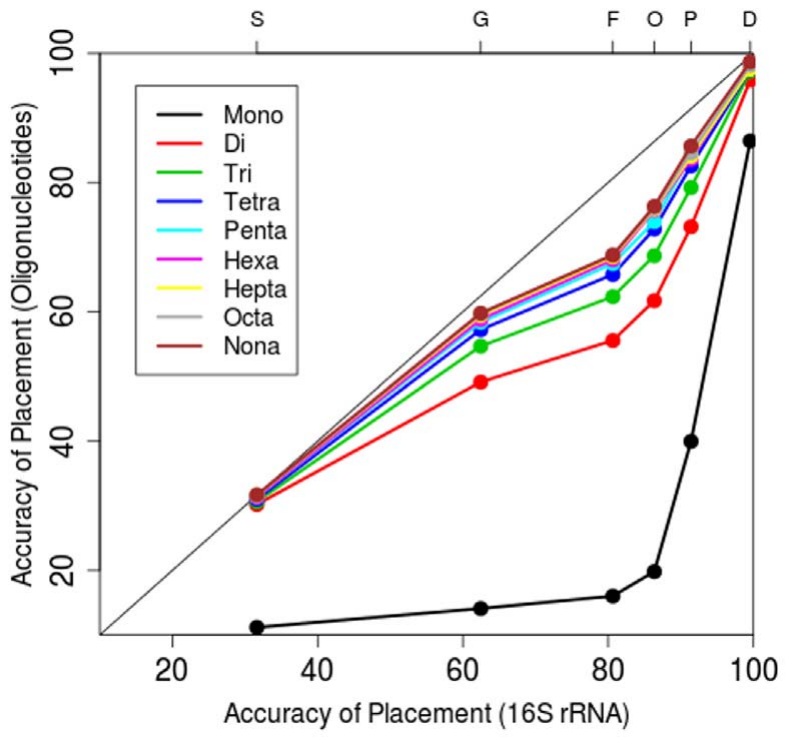
Oligonucleotide vs. 16S rRNA Comparisons. The ability place phylogenetically similar organisms together on a cladogram using mononucleotide through nonanucleotide signatures was tested against a cladogram generated using 16S rRNA for 1,424 completed prokaryotic genomes. This figure shows the percentage correct cladogram placement for oligonucleotide signature (x-axis) verses the percentage of correct cladogram placement for 16S rRNA (y-axis). Taxonomic level is show along top axis using: same species (S), same genus (G), same family (F), same phylum (P) and same domain (D). Mononucleotides through nonanucleotide signature trend lines are color-coded (see figure legend).

16S rRNA-based phylogenies are currently the gold standard for determining taxonomic relationships across the tree of life. Therefore, we focused on comparing oligonucleotide-based cladograms from mono- to nona- word lengths to a 16S rRNA-based phylogenetic tree of the 1,424 prokaryotes available from NCBI’s microbial genome database. For these comparisons, oligonucleotide and 16S based cladograms were analyzed by calculating the percentage of leaf nodes which contain nearest neighbors of the same taxonomic level. The percentage of nearest neighbors with the same taxonomy from oligonucleotide signatures (y-axis) is plotted relative to 16S rRNA (x-axis) for mononucleotide through nonanucleotide signatures (Figure 2) (data provided in Table S1). This analysis includes all major taxonomic levels with the top axis denoting taxonomic levels as: same species (S), same genus (G), same family (F), same order (O), same phylum (P) and same domain (D). A 1∶1 line shows the region of the plot with equivalence in performance between oligonucleotide word usage and 16S rRNA, and deviations from this 1∶1 line indicate that one method is outperforming the other. Notably, di- through nona- nucleotide signatures perform nearly as well as 16S rRNA when placing genomes of the same species and domain together on a cladogram, but are outperformed by 16S rRNA at clustering genomes in the genus through phylum levels together. As is evident in Figure 2, the placement of same taxon organisms together improves substantially as oligonucleotide word length increases from mono- through tetra-, followed by more gradual increases as word lengths are extended further. While these data substantiate the use of tetranucleotide frequency analysis as fast and effective way to assign taxonomy they also suggest that longer word length analyses can indeed provide better taxonomic resolution. It is again important to note that the lack of multiple organisms from all taxonomic levels makes it impossible to place nearest neighbors together for all cases.

**Figure 3:**
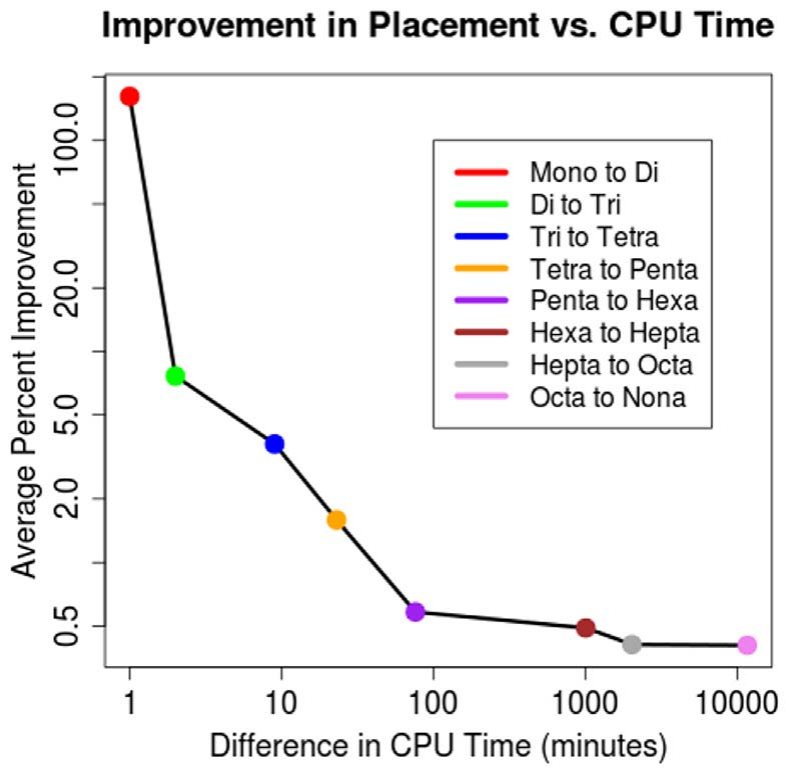
Improvement in Placement vs. CPU Time. The sum total percent improvement in placing identical taxonomic levels together on a cladogram as oligonucleotide length is increased verses the increase in CPU time required to calculate all Euclidean distances between 1,424 genomes. CPU time increases are due to the exponential increase in signature bins (and therefore variables in Euclidean distance calculations) as oligonucleotide lengths increase.

The downside of longer word length analyses is that computational time increases dramatically with longer oligonucleotide signatures, as the number of bins involved in calculating Euclidean distances increases as 4^(word length)^. Table S1 shows the CPU time required (running on a single 2.1 GHz core) to complete the Euclidean distance calculations and shows that, above word lengths of nine nucleotides, computational time quickly becomes intractable on modern computing hardware. Additionally, beyond word lengths of seven nucleotides the increase in CPU time does not correspond to a sizeable increase in prediction accuracy (Figure 3). This suggests a compromise between accuracy and computing time at the heptanucleotide length that is both effective at grouping taxa and well within the computational capabilities of computational genomicists. We focus the analyses below on comparisons between heptanucleotide and tetranucleotide signatures, while including other word length analyses in the Supporting Information.

**Figure 4:**
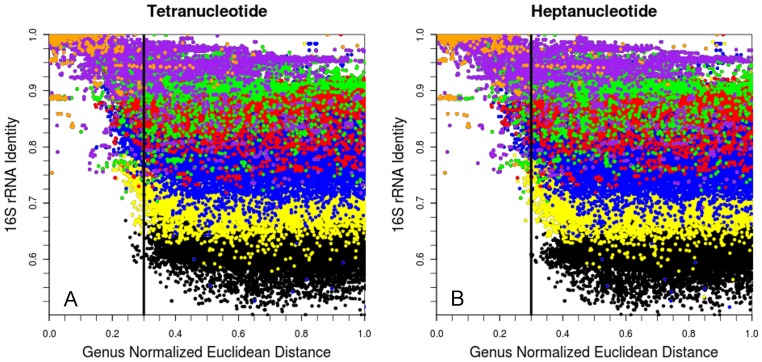
Tetranucleotide & Heptanucleotide vs. 16S rRNA identity. Plot of 16S percent identity verses genus normalized Euclidean distance for tetranucleotide (A) and heptanucleotide (B) signatures. Plots are colored based on the highest shared taxonomic level of the two organisms being compared: same species are in orange, same genus (purple), same family (green), same order (red), same phylum (blue), same domain (yellow) and different domain (black). Vertical lines added at a Euclidean distance of 0.3 for visual reference. By plotting 16S identity verses Euclidean distance this plot demonstrates the range of oligonucleotide Euclidean distances useful for discerning the taxonomic relationships between sequences. Additionally, this plot shows that low oligonucleotide Euclidean distances are a strong indicator that sequences are from phylogenetically close organisms.

Pairwise comparisons of 16S rRNA and oligonucleotide signatures were used to investigate how oligonucleotide Euclidean distances correlate with 16S rRNA identity. This was done directly by plotting Euclidean distance verses 16S rRNA percent identity for all organism pairs in our 1,424 member dataset (2,027,776 total points/comparisons). Figure 4 shows the corresponding plots for tetranucleotide and heptanucleotide signatures while Figure S3 shows plots over the range of mono- through nona- oligonucleotide signatures. Plots are colored based on the highest shared taxonomic level of the two organisms being compared: same species are in orange, same genus (purple), same family (green), same order (red), same phylum (blue), same domain (yellow) and different domain (black) (note that the plots are normalized so the largest genus to genus Euclidean distance is assigned a genus normalized Euclidean distance of 1.0 (Normalization factors in Table S2) Additionally, plots are truncated to this distance – due to their long tails. Figure 4 shows two very intriguing regions which are devoid of points located at high Euclidean distance/high 16S rRNA identity (upper right) and low Euclidean distance/low 16S rRNA identity (lower left). These regions contain rough “slopes” which naturally constrain the Euclidean distance/16S rRNA identity space occupied and allow estimation of the minimum and maximum Euclidean distances that bound different taxonomic level. Both plots in Figure 4 show a region at low Euclidean distances where most points are either same species or same genus (left of vertical lines). The existence of this region is a key for using oligonucleotide signatures as a method for identifying genomic or metagenomic fragments based on oligonucleotide signatures; points that fall into this region can be placed into a genus or species classification with a high probability.

The plots in Figure 4 also demonstrate that at greater Euclidean distances (to the right of the vertical line) it becomes increasingly difficult to correctly identify taxonomy, as the higher taxonomic levels blur together. This “blurring” explains why while oligonucleotide signatures perform nearly equivalently to 16S RNA for placing genus and species while their ability to differentiate higher taxonomic levels drops off significantly. The distinguishing feature between the tetranucleotide and heptanucleotide plots is the Euclidean distance where these dividing lines are located: in the heptanucleotide plot the line is shifted to the right, indicating greater potential for classifying genomic or metagenomic fragments at the species and genus levels. Note that the heptanucleotide plot shows a shallower negative slope in the lower-left region as compared to the tetranucleotide plot, which hints at more Euclidean distance space which is usable for disseminating higher taxonomic levels (family, order, phylum and domain) as the result of this shallower slope is less blurring of the higher taxonomic levels. This may be the reason why longer oligonucleotide signatures are slightly better than tetranucleotide signatures for correctly placing the higher taxonomic levels together.

**Figure 5:**
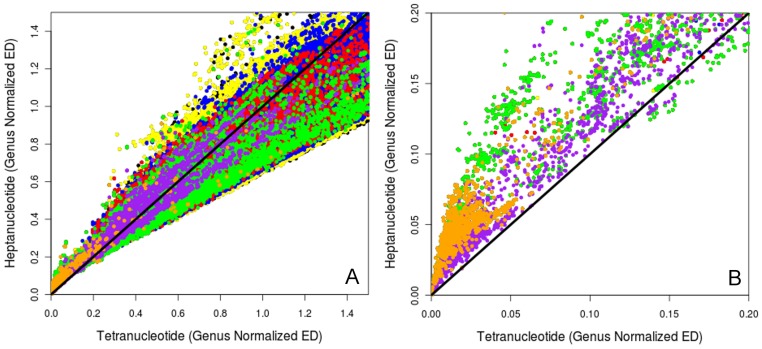
Tetranucleotide vs. Heptanucleotide. Plot of tetranucleotide verses heptanucleotide Euclidean distance for 1,424 genomes to a genus normalized Euclidean distance of 2.0 (A) and 0.20 (B). Plots are colored based on the highest shared taxonomic level of the two organisms being compared: same species are in orange, same genus (purple), same family (green), same order (red), same phylum (blue), same domain (yellow) and different domain (black). Plots include a 1∶1 line to mark equivalence between tetranucleotide and heptanucleotide Euclidean distances. These plots demonstrate lower Euclidean distance for closely related organisms (same genus/species) from heptanucleotide signatures, while moving towards shorter Euclidean distances for distantly related organisms from tetranucleotide signatures.

To directly compare tetranucleotide and heptanucleotide signatures we took the Euclidean distances for all pair-wise comparisons between all organisms for tetranucleotide and heptanucleotide signatures and plotted them against each other (Figures 5A and 5B). Figure 5A shows all points up to a genus normalized Euclidean distance of 1.0 (as in Figure 4) while Figure 5B is enlarged to show points near the origin. Plots use the same coloring-by-shared-taxa as per Figure 3, and include a 1∶1 line to show equivalent performance in grouping like taxa together. Figure 5A indicates that heptanucleotide signatures are producing relatively larger Euclidean distances for closely related organisms while performing equivalently to tetranucleotide signatures for distantly related organisms. This tends to stretch the same species and same genus portions of the Euclidean distance space while not affecting the domain and phylum regions. Focusing on the same species and same genus comparisons (orange and purple) in Figure 5B we see that almost all these points are above the 1∶1 line, indicating that the stretching in this region for heptanucleotide signatures is potentially very useful for placing same genus and same species together based on Euclidean distance. Additionally, these plots suggest the use of tetranucleotide signatures for comparisons between higher taxonomic levels (i.e. between phylums) as these points are mainly on the tetranucleotide side of the 45^o^ line. This raises an important point: different oligonucleotide word lengths might provide advantages in assigning different taxonomic levels. For instance, tetranucleotide analysis may indeed outperform heptanucleotide analysis when applied to a metagenomic dataset with many novel/unassignable species, where the focus might instead be on assigning reads at the phylum or domain levels.

**Figure 6:**
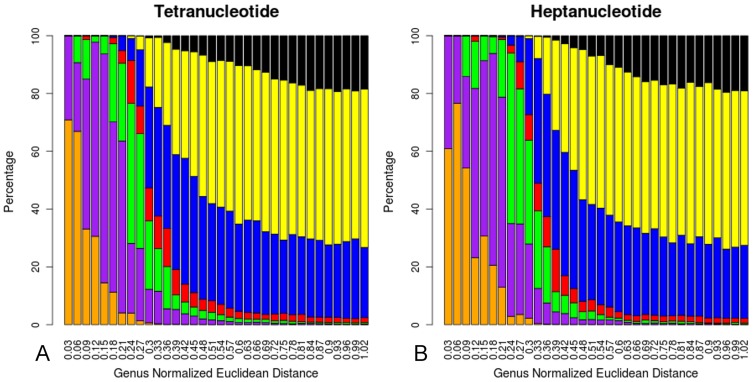
Leave-one-out Histograms. Histograms show the results of a leave-one-out analysis where the oligonucleotide-based Euclidean distance was calculated between all organisms (except self comparisons) and the percentage of organism matches which contain identical taxonomy for tetranucleotide (A) and heptanucleotide (B) signatures was binned based on genus normalized Euclidean distance. Plots are colored based on the highest shared taxonomic level of the two organisms being compared: same species are in orange, same genus (purple), same family (green), same order (red), same phylum (blue), same domain (yellow) and different domain (black). These plots are useful for determining the statistical likelihood of taxonomic matches between unknown sequences, as the percentages can be used to determine likelihood of a taxonomic match when the Euclidean distance between two unknown sequences has been calculated.

To further investigate of the probability of oligonucleotide signatures providing correct taxonomic information based on Euclidean distance, we devised a leave-one-out analysis where taxonomic assignment of one “unknown” organism (the “one left out”) was made by comparing oligonucleotide signatures with the other N-1 genomes. Thus N-1 total comparisons were made and binned based on their Euclidean distance, with results shown in Figure 6 (for visualization, the genus normalized Euclidean distance range was divided into 30 bins). Bins were plotted as stacked bars showing the percentages of similarity at each taxonomic level between all N-1 comparisons. Figure 6 plots tetranucleotide and heptanucleotide signatures (di- through nona- in Figure S4). The bars are color-coded as: same species (orange), same genus (purple), same family (green), same order (red), same phylum (blue), same domain (yellow) and different domain (black). These plots reveal the useful range of Euclidean distances for taxonomic determinations by showing which Euclidean distances have a high likelihood for correctly identifying the taxonomy of an unknown DNA sequence. These charts combine the information we had previously seen into a form which allows us to see point density based on taxonomy. For example, if we have two DNA sequences with a heptanucleotide-based Euclidean distance of 0.4 we would predict an approximately 45% chance they are within the same species or genus and a greater than 95% chance they are within the same family.

These bar charts validate what we had previously observed by showing the range of Euclidean distances corresponding to same species and same genus comparisons being spread out, while the tetranucleotide plot has taller same-genus and -species bars at short Euclidean distances, which then drop off at larger distances. This means that while longer word lengths may allow for more resolution the signal is getting mixed in with the Euclidean distances corresponding to higher taxonomic levels. While we are able to use longer word lengths for the purposes of differentiating between sequences, longer word lengths are less useful when trying to assign an unknown sequence based solely on a Euclidean distance. However, increased resolution will likely result in a substantial increase in the usefulness of oligonucleotide signatures, as other methods, such as NBCI BLAST, exist for direct comparisons between two DNA sequences.

**Figure 7:**
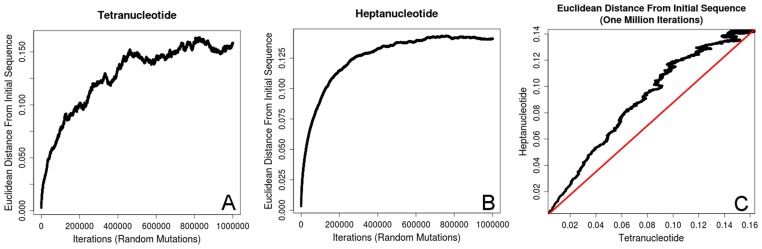
Random Mutations. This figure shows how a one million base pair DNA sequence responds to random mutations. Euclidean distance from the initial sequence is plotted for tetranucleotide (A) and heptanucleotide (B) verses iteration number. Figure 7C shows tetranucleotide verses heptanucleotide Euclidean distance by iteration with a 1∶1 line (red) to show equivalence. These plots show that heptanucleotide signatures demonstrate a faster increase in Euclidean distance from small changes in the DNA sequence, compared to tetranucleotide signatures, while leveling off and responding little to changes beyond approximately 600,000 iterations. Conversely, tetranucleotide signatures demonstrate smaller increases in Euclidean distance as a result of small perturbations in the DNA sequence, but continue to fluctuate to one million iterations.

We analyzed the degree to which tetranucleotide and heptanucleotide signatures respond to random mutations in a one million base pair DNA sequence. For this we took a randomly generated DNA sequence and randomly mutated a single base over one million iterations to measure the change in Euclidean distance when compared to the original sequence. The results of plotting Euclidean distance verses iteration number are in Figures 7A and 7B, while Figure 7C shows the Euclidean distances for tetranucleotide (x-axis) verses heptanucleotide (y-axis). This analysis shows that heptanucleotide signatures respond faster to small changes in the DNA sequence, confirming that they are better for differentiating between very similar sequences. Additionally, heptanucleotide curve shows saturation at approximately 600,000 mutations while the tetranucleotide curve continues to fluctuate to 1 million iterations. This is likely a product of the additional bins in the heptanucleotide analysis smoothing out the curve. As this analysis fails to reach large Euclidean distances away from the initial sequences it reinforces the idea that oligonucleotide signatures are not the result random mutations, as randomly mutating a sequence does nothing more that redistribute the bases into a different random pattern, while being unable to generate the strong biases in oligonucleotide usage seen in nature.

**Figure 8:**
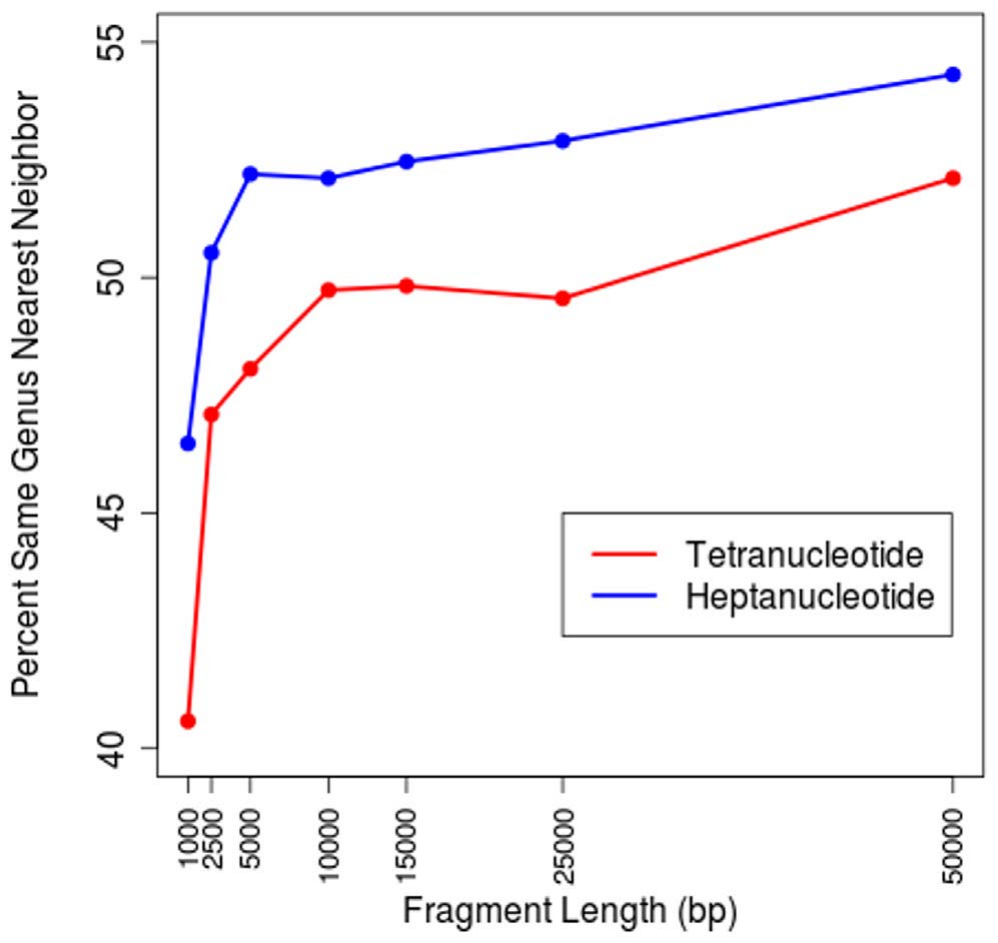
Metagenomic Sized Fragments. Completed prokaryotic genomes were broken into metagenomically relevant fragments sizes of: 1,000 bp, 2,500 bp, 5,000 bp, 10,000 bp, 15,000 bp, 25,000 bp and 50,000 bp by extracting a random fragment of each length from each of the 1,424 genomes. The tetranucleotide and heptanucleotide based Euclidean distance was calculated between each fragment and these distances were used to construct cladograms. Each cladogram was analyzed for the percentage of organisms with a nearest neighbor belonging to the same genus and this percentage is plotted verses fragment length. Improvement is seen as fragment length is increased, but the improvement levels off at approximately 10,000 bp for tetranucleotide signatures and approximately 5,000 bp for heptanucleotide signatures, with heptanucleotide signatures are performing better at all fragment lengths.

Oligonucleotide signatures have most often been applied to metagenomics datasets [18], [20], [24]–[26]. Within these analyses oligonucleotide signatures were implemented as a method for internally clustering short DNA fragments. This was accomplished by clustering fragments with similar oligonucleotide biases and using these clusters as the basis for further analyses, including assembly [18], [24]. To complement this work we investigated the relative usefulness of heptanucleotide signatures compared to tetranucleotide signatures as the basis for analyzing short DNA fragments. To test this we extracted fragments of metagenomically relevant lengths (1,000 bp, 2,500 bp, 5,000 bp, 10,000 bp, 15,000 bp, 25,000 bp and 50,000 bp) from the completed genome dataset, giving 1,424 genome fragments for each length. The tetranucleotide and heptanucleotide signatures for each fragment were calculated along with the Euclidean distance between each fragment. A distance matrix and cladogram were then generated from each fragment length group and nearest neighbor comparisons were completed, as done with whole genomes. Figure 8 shows the percentage of fragments belonging to the same genus which occur as nearest neighbors on the cladogram verses fragment length. As fragment length increases the prediction ability increases, although the increase is gradual beyond an initial spike at short fragment lengths. Interestingly, heptanucleotide signature improvement levels off at shorter fragment length (approximately 5,000 bp) while tetranucleotide signatures are not leveling off until approximately 10,000 bp. Also, we note that heptanucleotide signatures are better in all cases and the improvement in moving from tetranucleotide to heptanucleotide signatures allows 5,000 bp fragments to be placed with a level of accuracy not obtained until 50,000 bp using tetranucleotide signatures. At 50,000 bp the accuracy is below the levels obtained with whole genomes (52% vs. 57% for tetranucleotide and 54% vs. 59% for heptanucleotide), but these analyses indicate the usefulness of applying these methods to metagenomically relevant sequence lengths as well as the improvement due to using longer word lengths.

To study the fragment length required to overcome intrinsic oligonucleotide signature differences we broke the genomes of six organisms from six phyla (Escherichia coli, Mycoplasma leachii, Prochlorococcus marinus, Roseiflexus castenholzii, Sulfolobus islandicus and Thermotoga petrophila), plus a random 1.6 million base pair sequence into chunks in a range of lengths that are typical of metagenomic sequencing reads and scaffold assemblies (500 bp, 1,000 bp, 2,500 bp, 5,000 bp, 10,000 bp, 15,000 bp, 20,000 bp and 50,000 bp). We then calculated the average Euclidean distance between all organisms (including self comparisons) for all fragment lengths using tetranucleotide (Figure S5) and heptanucleotide (Figure S6) signatures. By plotting fragment length verses Euclidean distance for all organisms we can see that the 10,000 base pair fragments demonstrate the minimum ideal fragment size required to differentiate between organisms from different phyla, although fragments as short as 2,500 base pair where demonstrating some ability for differentiation. Additionally, it is important to note the Euclidean distances in the self comparisons as these distances show the differences in oligonucleotide signatures found in different regions of a complete genome. While it is clear the average overall oligonucleotide signature for an organism is evolutionarily conserved newly integrated portions may not be mutated sufficiently to display the biases in which these methods rely. As a result, this study shows that oligonucleotide analysis is only useful on approximately 10,000 bp or larger fragments.

Using a 10,000 base pair minimum fragment lengths we ran a comparison between tetranucleotide and heptanucleotide signatures ability to correctly assign fragments from metagenomics data using the NCBI non-redundant (nt) database. As a relevant sample set we analyzed all scaffolds over 10,000 base pairs in length from the five sampling locations within the Bison Pool metagenomics dataset [18]. For an accurate comparison NCBI BLAST was used for the determination of the “correct” sequence match in the nt database. Results were parsed to the genus level and Table S3 shows the best BLAST matches for the Bison Pool scaffolds along with the best tetranucleotide and heptanucleotide matches. We calculated the percentage of hits in which tetranucleotide and heptanucleotide signatures agree with the genus match from NCBI BLAST and found that tetranucleotide signatures agree 39.1% of the time while heptanucleotide signatures agree 41.9% of the time. While this is not a huge improvement it does show that heptanucleotide signatures are the better choice when assigning taxonomic labels to metagenomic data. Additionally, many of these hits had 2^nd^ or 3^rd^ best hits from different genera. This is the case for both the oligonucleotide based hits and the BLAST hits, so the percentages from these “best hit” comparisons are likely artificially low.

To investigate the effect scaffold length has on Euclidean distance for a dataset of metagenomic scaffolds we calculated the tetranucleotide and heptanucleotide Euclidean distances between scaffolds and related sequences in the NCBI nt database. This analysis randomly sampled 242 scaffolds (with lengths ranging from 221 bp to 13,363 bp) from twenty five metagenome projects [33] that encompass a wide range of geochemically diverse environments–and, ostensibly, taxonomically diverse communities–collected within Yellowstone National Park (listed in Table S4),. NCBI BLAST was first used to identify DNA sequences within the NCBI nt database that showed homology to these 242 metagenome scaffolds. Subsequently, tetranucleotide and heptanucleotide Euclidean distances were calculated between these 242 metagenome scaffolds and all their nt homologs (resulting in 5,840 total comparisons). Plots of scaffold length verses Euclidean distance for tetranucleotide (Figure S7-A) and heptanucleotide (Figure S7-B) signatures show that short scaffolds have relatively long Euclidean distances to their homologs. This is especially evident in the “clean” region at the lower left of the heptanucleotide plot (Figure S7-B). Interestingly, tetranucleotide signatures are able to obtain low genus normalized Euclidean distances (<1.0) from short (<4,000 bp) DNA fragments while heptanucleotide signatures do not.

Figure S8 shows a plot of tetranucleotide Euclidean distance verses heptanucleotide Euclidean distance which has been colored to indicated scaffold length as: less than 800 bp (red), 800 bp to 1,000 bp (blue), 1,000 bp to 2,000 bp (green), 2,000 to 5,000 bp (orange) and over 5,000 bp (black). This plot demonstrates the consistency of heptanucleotide Euclidean distances between fragments of similar length while those fragments are spread across a large range of tetranucleotide Euclidean distance space. This plot also reinforces the observation that longer scaffolds (>5,000 bp) are required to obtain normalized heptanucleotide Euclidean distances below 1.0, whereas all scaffold lengths are capable of obtaining short tetranucleotide Euclidean distances. Additionally, the “banding” of colors along the heptanucleotide axis confirms the relationship between scaffold length and Euclidean distance between similar sequences when using heptanucleotide signatures. These minimum Euclidean distances must be accounted for when using oligonucleotide signatures with real metagenomics datasets as they set the standard for determining the relatedness of two scaffolds.

Twenty five Yellowstone National park metagenomes (Table S4) were analyzed to determine the frequency of large scaffolds, as these are the most useful for taxonomic binning applied to real metagenomics datasets. Figure S9 shows a histogram of the average frequency of large (>10,000 bp) scaffolds within these metagenomes. While the majority of the larger scaffolds are 10,000 bp to 15,000 bp, many are longer, with each of the metagenomes averaging seven scaffolds over 50,000 bp. The data include a total of eighty-seven large scaffolds, although it must be noted that next generation sequencing becomes more affordable, the frequency of large scaffolds in datasets will continue to increase, resulting in the increased applicability of these methods.

Our analyses show that longer oligonucleotide signatures have great applicability for homology binning and taxonomic identification. In many cases oligonucleotide signatures were able to compete with 16S rRNA for resolving taxonomy, demonstrating the usefulness of oligonucleotide signatures as for resolving the taxonomic source of a DNA fragment, an increasingly important challenge as environmental sequencing becomes the norm in how DNA/RNA is obtained from complex communities. The sometimes substantial improvements in taxonomic resolution gained from analyzing longer oligonucleotide word lengths comes at a fairly cheap computational cost, and we call into question whether the current paradigm of tetranucleotide word length analysis in metagenomics should undergo a much needed shift.

## Methods

A complete set of non-draft sequenced microbial genomes (including 1,315 bacteria and 109 archaea) (Table S5) were downloaded from the National Center for Biotechnology Information (NCBI) website (ftp://ftp.ncbi.nih.gov/genomes/Bacteria/) on June 21^st^, 2012. The genomes were filtered to remove plasmids to allow for an analysis of only chromosomal DNA. Additionally, 16S rRNA sequences for all included genomes were downloaded from the Ribosomal Database Project (RDP) (http://rdp.cme.msu.edu/). All genomes included in this study contain taxonomic information obtained from the NCBI taxonomic database (ftp://ftp.ncbi.nih.gov/pub/taxonomy/) and parsed to include: species, genus, family, order, phylum and domain annotations. An analysis of the phylums included in this study shows that while the NCBI genomes database contains good diversity (30 phylums) it also includes a strong bias towards proteobacteria (43% of total genomes) and firmicutes (19% of total genomes).

All chromosomes were analyzed to determine their mono- through nona- oligonucleotide signatures using a “sliding window” to find the count of each possible oligonucleotide combination [7]. These counts were converted into percentages where the ratio of all percentages represents the genetic signature. The Euclidean distance between chromosomes was determined using the following formula:
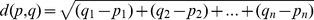



Where each (*p, q*) set represents a bin from the oligonucleotide signature. The Euclidean distances between all organism pairs were converted into a distance matrix for analysis using the neighbor program within the Phylip software package [34]. This resulted in cladograms representing the Euclidean distances (i.e. oligonucleotide signatures) between all members of the 1,424 organism dataset. Corresponding bacterial and archaeal 16S rRNA sequences were combined and aligned using the RDP’s on-line tools. The aligned 16S rRNA sequences where converted into a phylogenetic tree using the dnadist and neighbor tools within Phylip. The oligonucleotide signature based cladograms and the 16S rRNA based phylogenetic tree were analyzed using Bioperl’s TreeIO [35] tools to extract the distance between all leaf nodes. Results were filtered to generate a list of the nearest neighbor for all leaf nodes in all cladograms. Using taxonomy data for all leafs and their nearest neighbor we determined the percentage of occurrences when a nearest neighbor is from the same taxonomic group (i.e. same domain, same phylum, etc). Additionally, the taxonomic data between nearest neighbors allowed for the color-coding of cladogram nodes based on taxonomy.

Euclidean distance verses 16S identity plots were generated by plotting the Euclidean distance between all organism pairs in our 1,424 member dataset verses the 16S identity between the pair. The identity between aligned 16S rRNA sequences was determined using the dnadist program within Phylip. Taxonomy data was also included to color-code the plot. The genus normalized Euclidean distance normalization metric was derived from dividing all Euclidean distances by the largest genus-genus Euclidean distance for all oligonucleotide lengths (Table S2).

To generate the leave-one out analysis we calculated the Euclidean distance between all organisms in our 1,424 member dataset, not including self-comparisons. We then organized all resulting distances into thirty equally sized bins and calculated the taxonomical relationships for all organism pairs in each bin. Each bin was then analyzed for the percentage of times the same taxonomic identity was seen (i.e. how often a bin contained organism pairs from the same genus).

To determine random divergence we constructed a random one million base pair DNA sequence. The sequence was subjected to one million iterations where we randomly selected a single base and mutated it to a randomly selected base. The mutated DNA sequence was written to disk every one hundred iterations and each of these sequences were compared to the original by calculating the tetranucleotide and heptanucleotide signature and calculating the Euclidean distance from the original sequence. The results were plotted as iteration number verses Euclidean distance from the original sequence.

To analyze metagenomically relevant fragments from 1,424 completed genomes we randomly pulled 1,000, 2,500, 5,000, 10,000, 15,000, 25,000 and 50,000 base pair fragments from each completed genome and calculated tetranucleotide/heptanucleotide signatures for all fragments. The Euclidean distance was calculated between each fragment for all fragment lengths and each set of Euclidean distances was converted into a distance matrix. Distance matrices were analyzed using the neighbor application in Phylip to generate cladograms. BioPerl’s TreeIO was used to calculate the nearest neighbor for all nodes and the NCBI taxonomy was used to pull genus of all sequenced genomes included. The percentage of nearest neighbors having the same genus was calculated and plotted verses fragment length for both tetranucleotide and heptanucleotide signatures.

To determine the Euclidean distances based on fragment length the organism’s chromosome was broken in chunks with lengths of: 50,000, 20,000, 15,000, 10,000, 5,000, 2,500, 1,000 and 500 base pairs. The tetranucleotide and heptanucleotide signatures were calculated for all chunks along with the Euclidean distances between all chunks. The average Euclidean distance between all chunks was then calculated between all organism pairs over all chunk lengths. These average Euclidean distances were then plotted verses chunk length for each organism pair.

To determine nt database matches for the Bison Pool metagenome dataset using oligonucleotide signatures both the nt database and the Bison Pool dataset were parsed to DNA sequences in excess of 10,000 base pairs. Next, the tetranucleotide and heptanucleotide signatures were calculated for all sequences in the nt database as well as the Bison Pool dataset. The Euclidean distance was calculated between all members of the Bison Pool dataset and nt using both tetranucleotide and heptanucleotide signatures, with the pairing with the lowest Euclidean distance designated as the best match. NCBI BLAST was used between the over 10,000 base pair nt database and the over 10,000 base pair Bison Pool datasets to determine the “correct” best match between the metagenomes and the nt database. Results were then analyzed for how often the best BLAST hit and shortest Euclidean distance hit agreed.

The twenty-five Yellowstone National Park metagenomes were combined into a single file and parsed so that every 700^th^ sequence was pulled out for analysis, giving us 242 scaffolds. NCBI BLAST was used to find all related sequences for the 242 metagenomic scaffolds within the NCBI nt database. The tetra- and hepta- nucleotide Euclidean distance was calculated between all metagenomic scaffolds and all their related hits in the nt database. The calculated Euclidean distances and the scaffold lengths were plotted using R.

Perl scripts developed for the determination of oligonucleotide signatures from DNA sequences as well as for calculating the Euclidean distance between oligonucleotide signatures are available for download from the Raymond ground website at evolution.asu.edu.

## Supporting Information

Figure S1 **Tetranucleotide Signatures.** Bar chart showing the 256 bins possible for tetranucleotide signatures and how they are occupied by *Escherichia coli* (red), *Sulfolobus islandicus* (green) and a 1.6 million base pair random sequence (blue) – ordered high to low by percentage. *E. coli* and *S. islandicus* have biases towards specific bins while the random sequence occupies all bins relatively equally, as tetranucleotide words are randomly assigned. The non-random nature of DNA sequences from real organisms shows that nature is not random and this non-random nature can be exploited as an oligonucleotide signature.
(TIF)

Figure S2 **Cladograms Based on Oligonucleotide Signatures.** Cladograms derived from dinucleotide through nonanucleotide signatures using Euclidean distances between 1,424 sequenced microbes. Terminal branches are color-coded to depict nearest neighbor taxonomic relationships as: strong relationships (same species or same genus) in red, good relationships (phylum or better) in blue, same domain in yellow and different domain in black. This figure demonstrates that di- through nona- nucleotide signatures are able to correctly place taxonomically similar organisms together on a cladogram.
(TIF)

Figure S3 **Oligonucleotide Signatures vs. 16S rRNA identity.** Plot of 16S percent identity verses genus normalized Euclidean distance for mononucleotide through nonanucleotide signatures. Plots are colored based on the highest shared taxonomic level of the two organisms being compared: same species are in orange, same genus (purple), same family (green), same order (red), same phylum (blue), same domain (yellow) and different domain (black). These plots show that the Euclidean distance space useful for same species comparisons is enlarged as oligonucleotide length is increased, with the most noticeable increases occurring at shorter oligonucleotide lengths.
(TIF)

Figures S4 **Leave-one-out Histograms.** Histograms show, by genus normalized Euclidean distance, the percentage of organism matches which contain identical taxonomy for mononucleotide through nonanucleotide signatures. Plots are colored based on the highest shared taxonomic level of the two organisms being compared: same species are in orange, same genus (purple), same family (green), same order (red), same phylum (blue), same domain (yellow) and different domain (black). These histograms demonstrate the expansion of usable Euclidean distance space for making same genus and same species taxonomic identifications as oligonucleotide length increases.
(TIF)

Figure S5 **Variable Fragment Lengths Plots.** Figures S5 and S6 show the average tetranucleotide (S5) and heptanucleotide (S6) Euclidean distances between genome fragments of lengths between 500 bp and 50,000 bp for six organisms (Escherichia coli, Mycoplasma leachii, Prochlorococcus marinus, Roseiflexus castenholzii, Sulfolobus islandicus and Thermotoga petrophila), plus a random 1.6 million base pair. By plotting fragment length verses Euclidean distance for all organisms it can be seen that 10,000 base pair fragments demonstrate the minimum ideal fragment size required to differentiate between organisms from different phyla, although fragments as short as 2,500 base pair where demonstrating some ability for differentiation.
(TIF)

Figure S6 **Variable Fragment Lengths Plots.**
(TIF)

Figure S7 **Tetra- and Hepta- Nucleotide Euclidean Distance verses Scaffold Length.** These figures show tetranucleotide (A) and heptanucleotide (B) genus normalized Euclidean distance verses scaffold length for comparisons between 242 metagenomic scaffolds and all related sequences within the nt database. These figures demonstrate the Euclidean distances seem for a variety of scaffold lengths along with the possible variations in Euclidean distance for a given scaffold length.
(TIF)

Figure S8 **Tetranucleotide verses Heptanucleotide Euclidean Distances.** This figure shows the tetra- and hepta- nucleotide genus normalized Euclidean distances between metagenomic scaffolds and their related sequences within the NCBI nt database. Points are colored by scaffold length as: less than 800 bp (red), 800 bp to 1,000 bp (blue), 1,000 bp to 2,000 bp (green), 2,000 to 5,000 bp (orange) and over 5,000 bp (black). This plot is based on 242 scaffolds ranging in size from 221 bp to 13,363 bp and includes 5,840 comparisons to related sequences in the nt database.
(TIF)

Figure S9 **Histogram of Average Large Scaffold Counts in Metagenomic Datasets.** This figure shows a histogram of the average frequency of large (>10,000 bp) scaffolds across twenty-five metagenomic datasets collected within Yellowstone National Park. These metagenomic datasets average eighty-seven scaffolds over 10,000 bp, including seven which are over 50,000 bp.
(TIF)

Table S1 **Percentages from Nearest-Neighbor Analyses with CPU Time.** Mononucleotide through nonanucleotide signatures were compared with 16S rRNA using nearest neighbor prediction ability (the percentage of times taxonomically identical genomes were placed as nearest neighbors on a cladogram). This table includes the values determined from this calculation (Figure 2 shows these values graphically). Additionally, this table shows the CPU time required to calculate the Euclidean distances between all 1,424 organism pairs.
(XLS)

Table S2 **Largest Same Genus Euclidean Distance (Genus Normalized Euclidean Distance Normalization Factor).** Euclidean distances were normalized using the largest same genus distance for each oligonucleotide length. These normalizations were completed to correct for the shrinking of Euclidean distances as oligonucleotide lengths increased due to the additional bins, and the subsequently smaller percentages each bin contained.
(XLS)

Table S3 **Bison Pool Metagenome Best Hits Using Oligonucleotide Signatures and BLAST.** Scaffold over 10,000 base pairs in length were compared to the nt database using NCBI BLAST, tetranucleotide signatures and heptanucleotide signatures. This table shows: genus of best BLAST hit (with e-value to that hit), genus of best tetranucleotide signature hit (with Euclidean distance to that hit) and genus of best heptanucleotide signature hit (with Euclidean distance to that hit) for each Bison Pool scaffold.
(XLS)

Table S4 **Metagenomes Included.** This table lists the twenty-five Yellowstone National Park metagenomic sample sets used and their JGI/IMG designations.
(XLS)

Table S5 **Organism Names and Accession Numbers for All Included Genomes.** This table contains the organism name and NCBI accession number for 1,424 genomes used in this study. Table also contains the RDP accession number for the 16S rRNA sequence corresponding to the genomes used in this study.
(XLS)
