# Supplementary figures and images for: Resolving Prokaryotic Taxonomy without rRNA: Longer Oligonucleotide Word Lengths Improve Genome and Metagenome Taxonomic Classification
Source: PLoS One. 2013 Jul 1;8(7):e67337. doi: 10.1371/journal.pone.0067337 (PMC3698125; doi:10.1371/journal.pone.0067337)

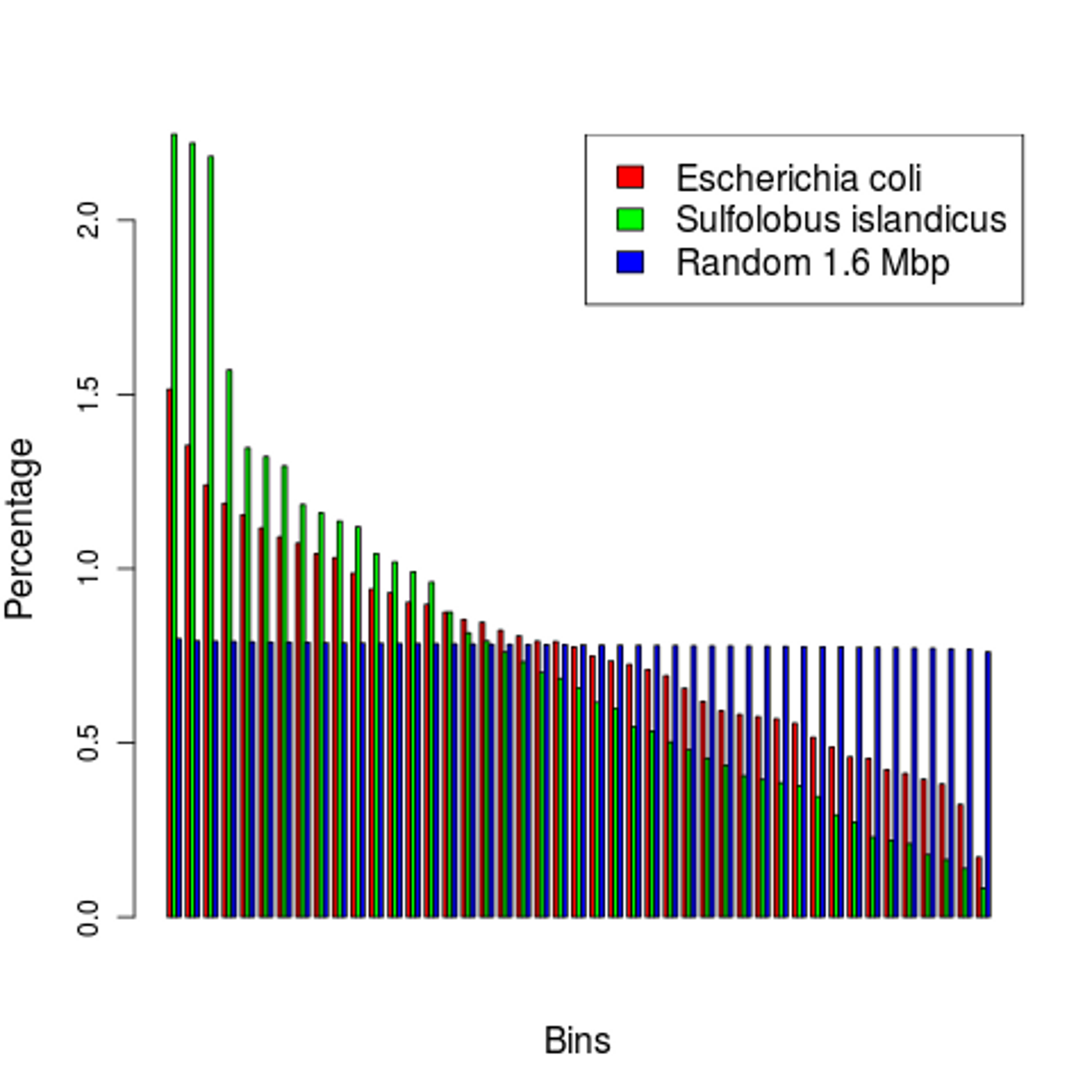

Supplement: Figure S1 — Tetranucleotide Signatures. Bar chart showing the 256 bins possible for tetranucleotide signatures and how they are occupied by Escherichia coli (red), Sulfolobus islandicus (green) and a 1.6 million base pair random sequence (blue) – ordered high to low by percentage. E. coli and S. islandicus have biases towards specific bins while the random sequence occupies all bins relatively equally, as tetranucleotide words are randomly assigned. The non-random nature of DNA sequences from real organisms shows that nature is not random and this non-random nature can be exploited as an oligonucleotide signature. (TIF) [file pone.0067337.s001.tif]

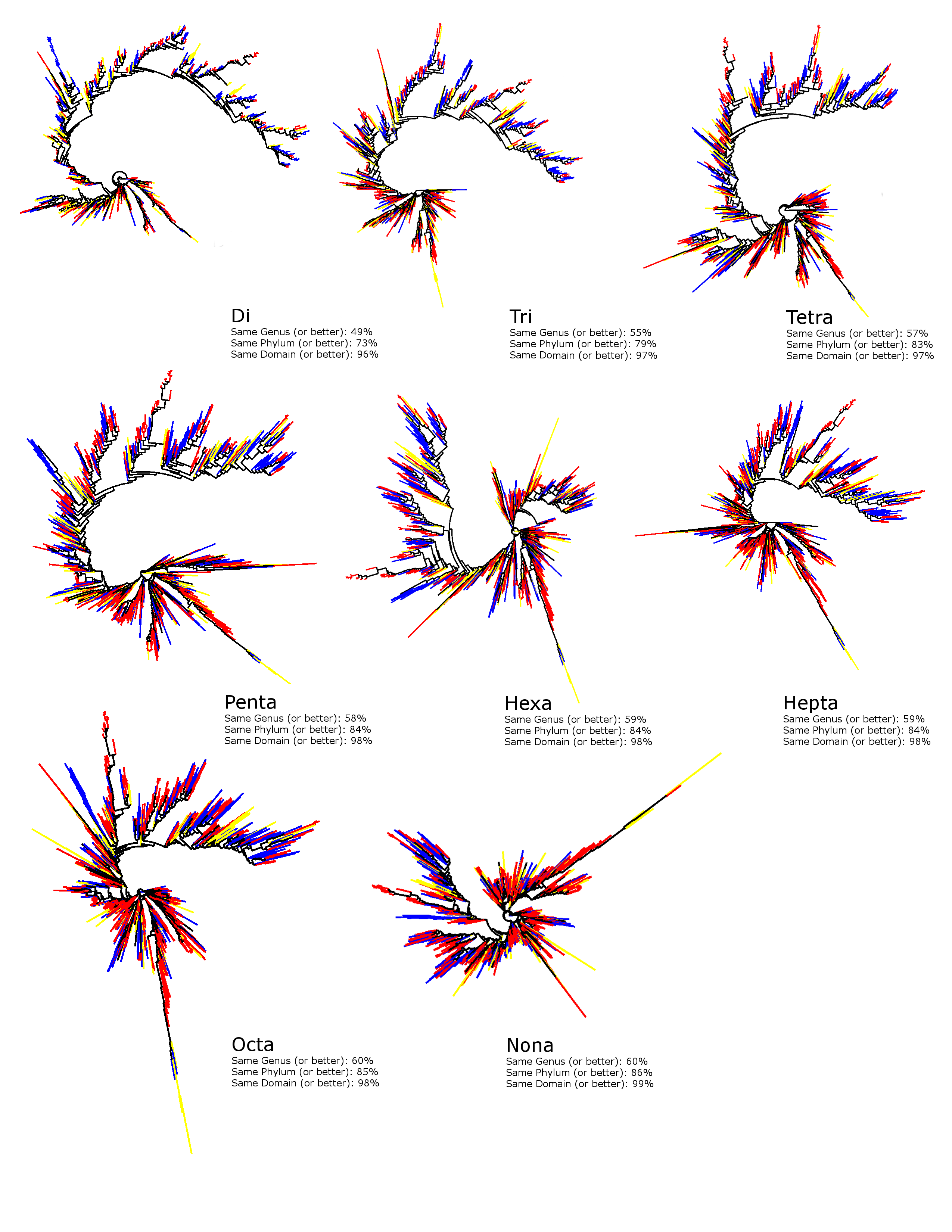

Supplement: Figure S2 — Cladograms Based on Oligonucleotide Signatures. Cladograms derived from dinucleotide through nonanucleotide signatures using Euclidean distances between 1,424 sequenced microbes. Terminal branches are color-coded to depict nearest neighbor taxonomic relationships as: strong relationships (same species or same genus) in red, good relationships (phylum or better) in blue, same domain in yellow and different domain in black. This figure demonstrates that di- through nona- nucleotide signatures are able to correctly place taxonomically similar organisms together on a cladogram. (TIF) [file pone.0067337.s002.tif]

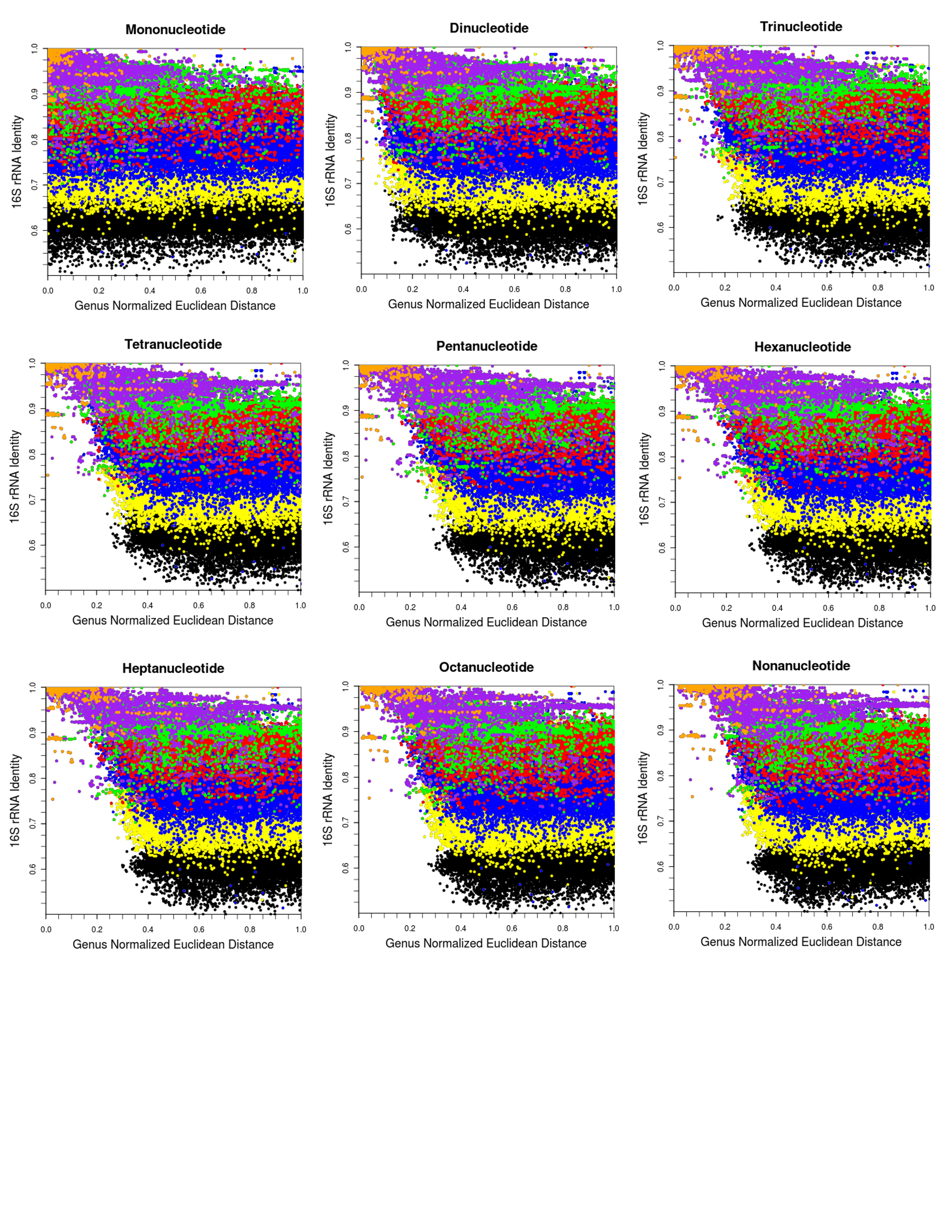

Supplement: Figure S3 — Oligonucleotide Signatures vs. 16S rRNA identity. Plot of 16S percent identity verses genus normalized Euclidean distance for mononucleotide through nonanucleotide signatures. Plots are colored based on the highest shared taxonomic level of the two organisms being compared: same species are in orange, same genus (purple), same family (green), same order (red), same phylum (blue), same domain (yellow) and different domain (black). These plots show that the Euclidean distance space useful for same species comparisons is enlarged as oligonucleotide length is increased, with the most noticeable increases occurring at shorter oligonucleotide lengths. (TIF) [file pone.0067337.s003.tif]

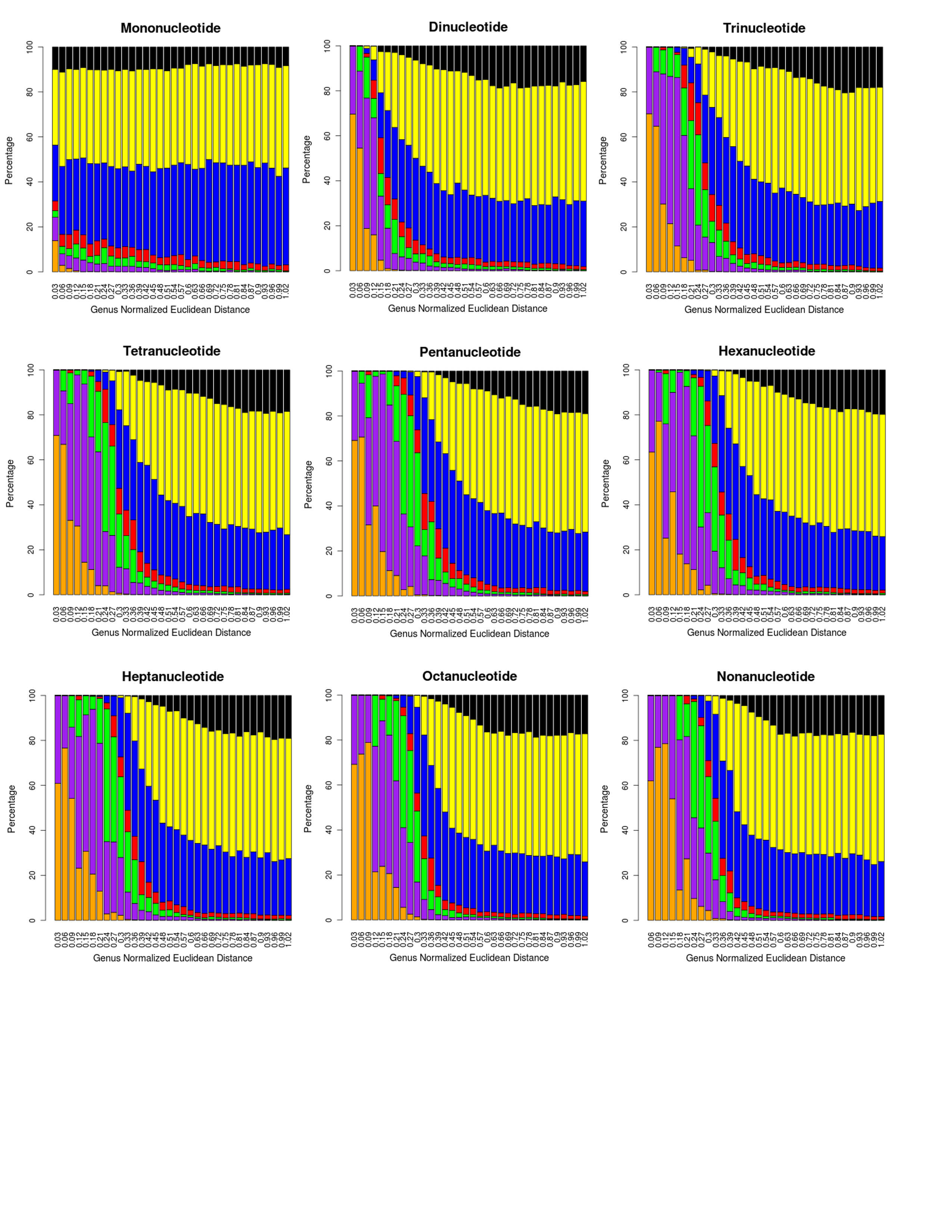

Supplement: Figures S4 — Leave-one-out Histograms. Histograms show, by genus normalized Euclidean distance, the percentage of organism matches which contain identical taxonomy for mononucleotide through nonanucleotide signatures. Plots are colored based on the highest shared taxonomic level of the two organisms being compared: same species are in orange, same genus (purple), same family (green), same order (red), same phylum (blue), same domain (yellow) and different domain (black). These histograms demonstrate the expansion of usable Euclidean distance space for making same genus and same species taxonomic identifications as oligonucleotide length increases. (TIF) [file pone.0067337.s004.tif]

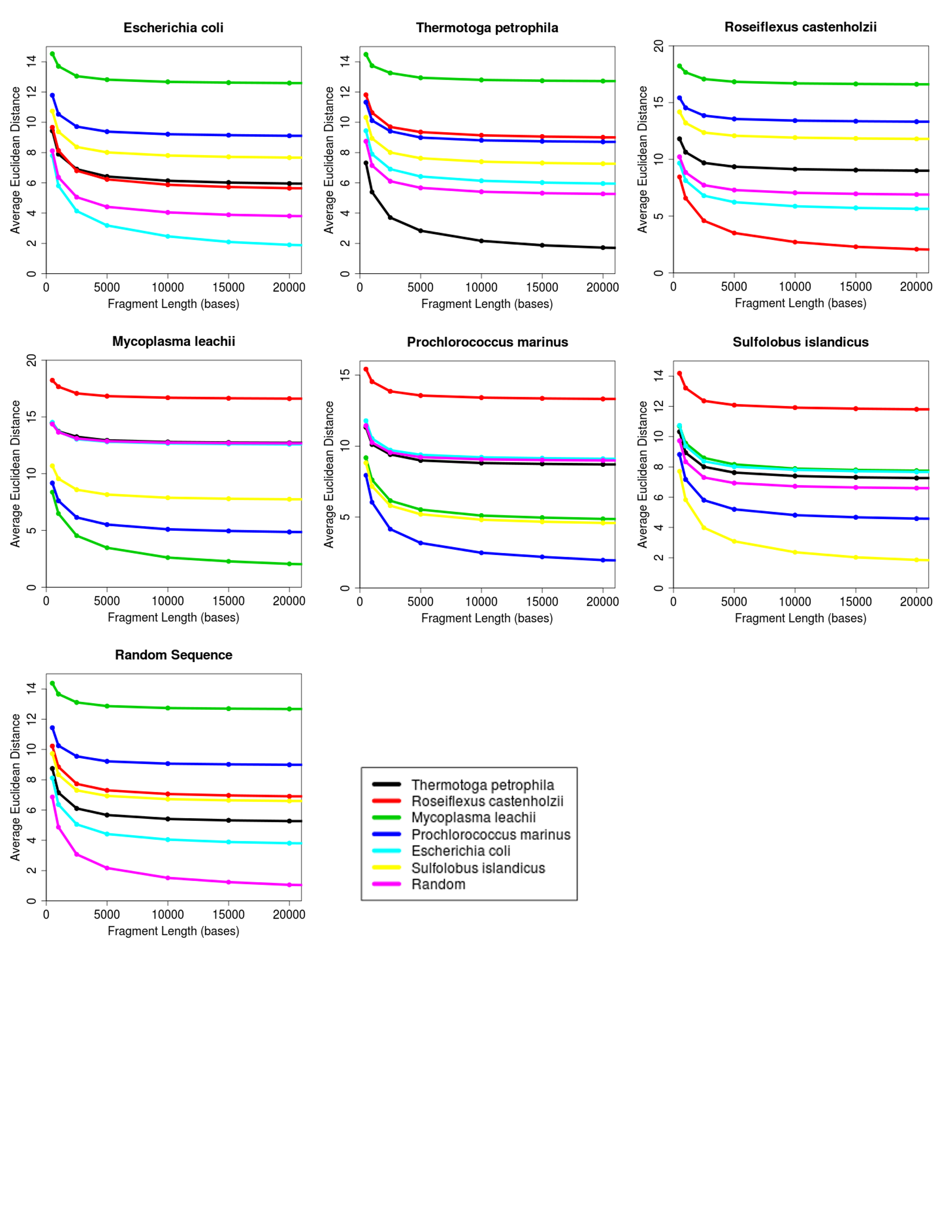

Supplement: Figure S5 — Variable Fragment Lengths Plots. Figures S5 and S6 show the average tetranucleotide (S5) and heptanucleotide (S6) Euclidean distances between genome fragments of lengths between 500 bp and 50,000 bp for six organisms (Escherichia coli, Mycoplasma leachii, Prochlorococcus marinus, Roseiflexus castenholzii, Sulfolobus islandicus and Thermotoga petrophila), plus a random 1.6 million base pair. By plotting fragment length verses Euclidean distance for all organisms it can be seen that 10,000 base pair fragments demonstrate the minimum ideal fragment size required to differentiate between organisms from different phyla, although fragments as short as 2,500 base pair where demonstrating some ability for differentiation. (TIF) [file pone.0067337.s005.tif]

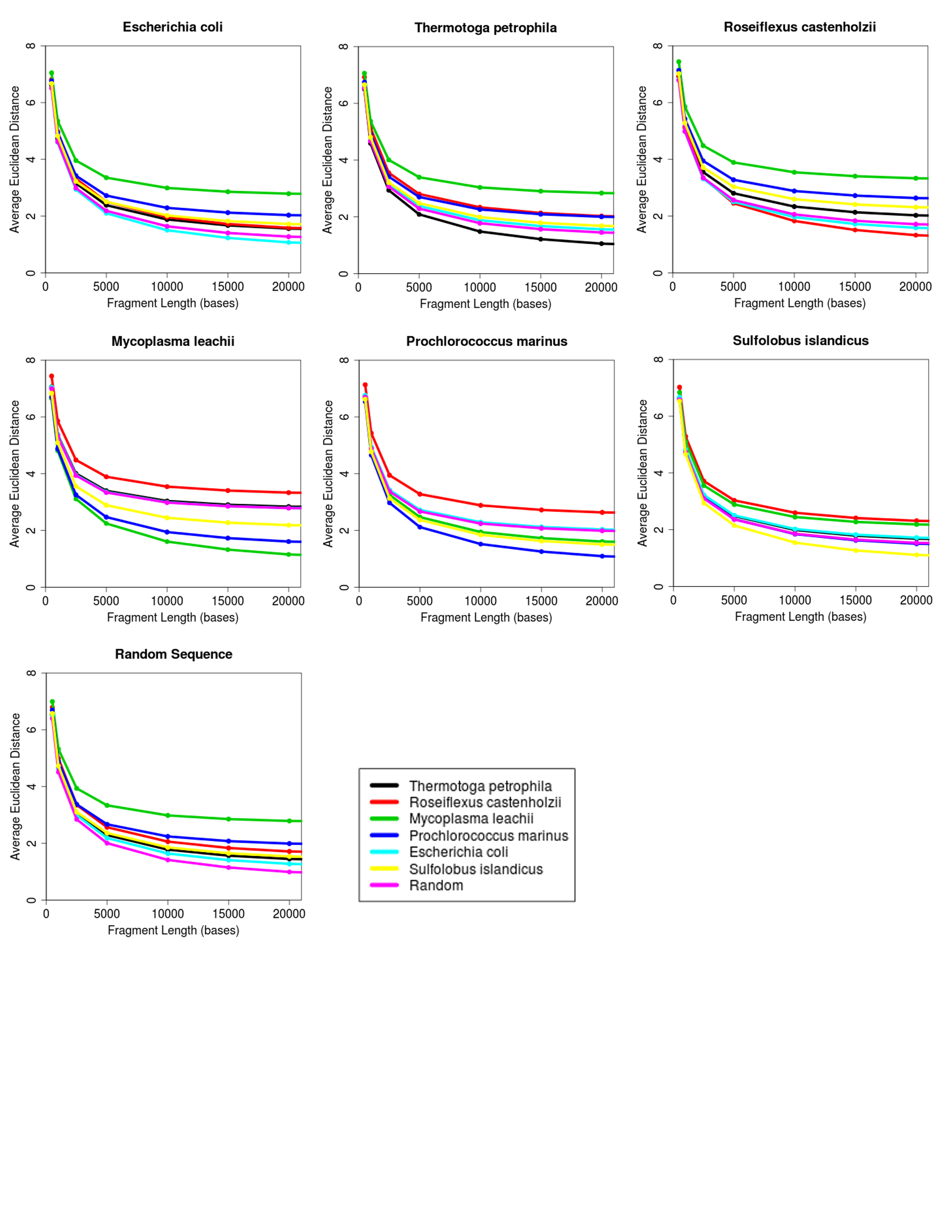

Supplement: Figure S6 — Variable Fragment Lengths Plots. (TIF) [file pone.0067337.s006.tif]

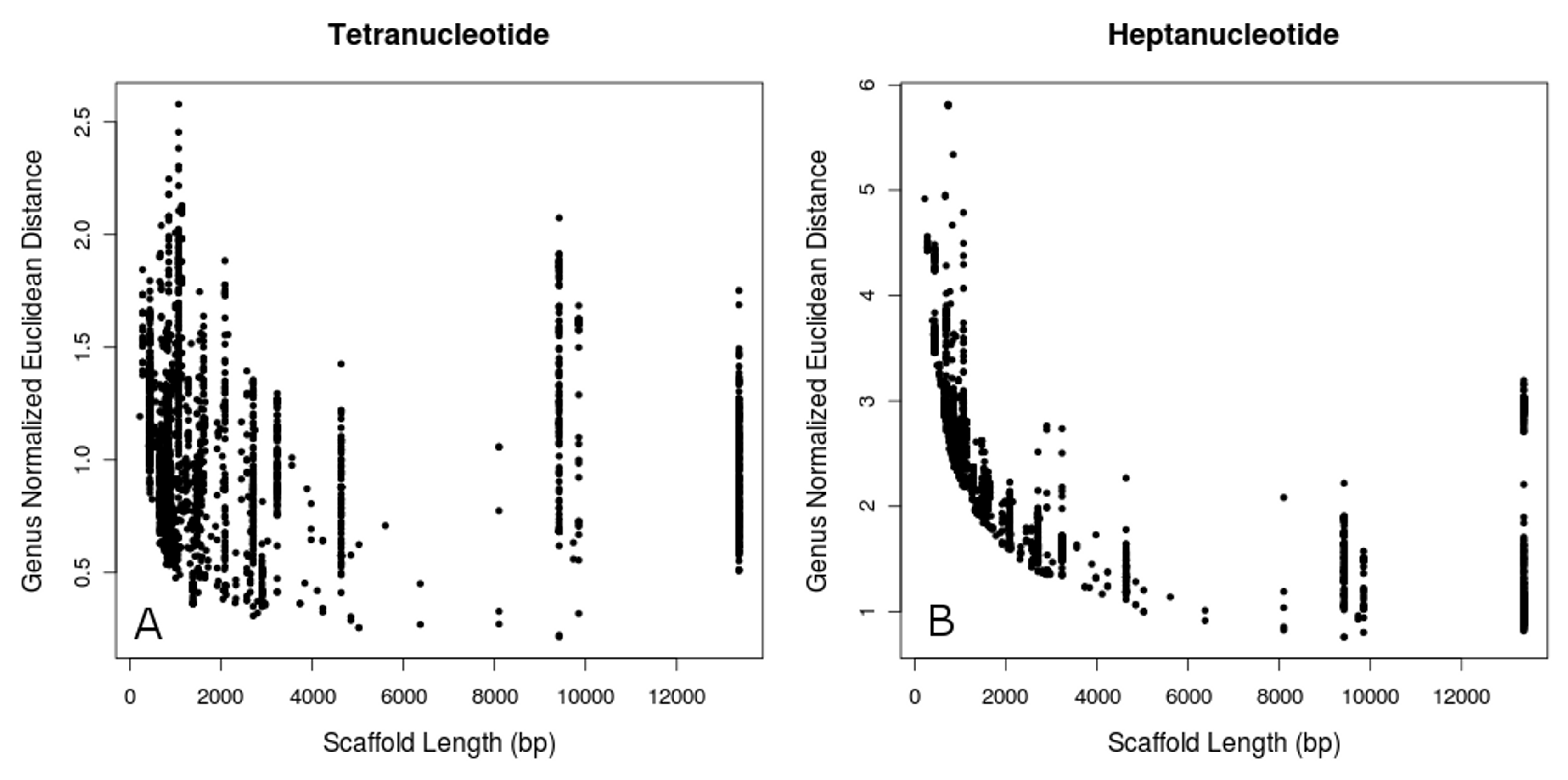

Supplement: Figure S7 — Tetra- and Hepta- Nucleotide Euclidean Distance verses Scaffold Length. These figures show tetranucleotide (A) and heptanucleotide (B) genus normalized Euclidean distance verses scaffold length for comparisons between 242 metagenomic scaffolds and all related sequences within the nt database. These figures demonstrate the Euclidean distances seem for a variety of scaffold lengths along with the possible variations in Euclidean distance for a given scaffold length. (TIF) [file pone.0067337.s007.tif]

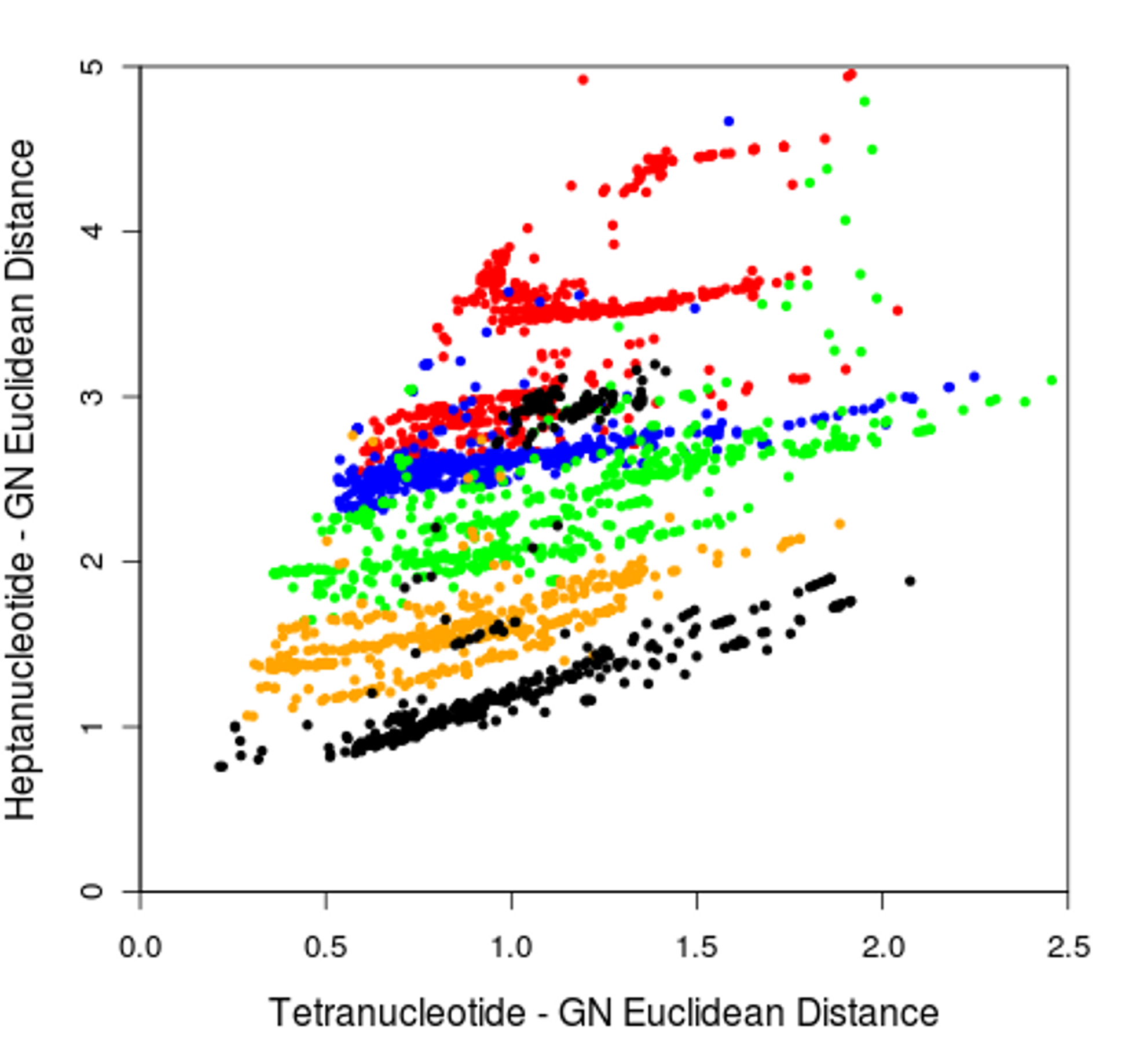

Supplement: Figure S8 — Tetranucleotide verses Heptanucleotide Euclidean Distances. This figure shows the tetra- and hepta- nucleotide genus normalized Euclidean distances between metagenomic scaffolds and their related sequences within the NCBI nt database. Points are colored by scaffold length as: less than 800 bp (red), 800 bp to 1,000 bp (blue), 1,000 bp to 2,000 bp (green), 2,000 to 5,000 bp (orange) and over 5,000 bp (black). This plot is based on 242 scaffolds ranging in size from 221 bp to 13,363 bp and includes 5,840 comparisons to related sequences in the nt database. (TIF) [file pone.0067337.s008.tif]

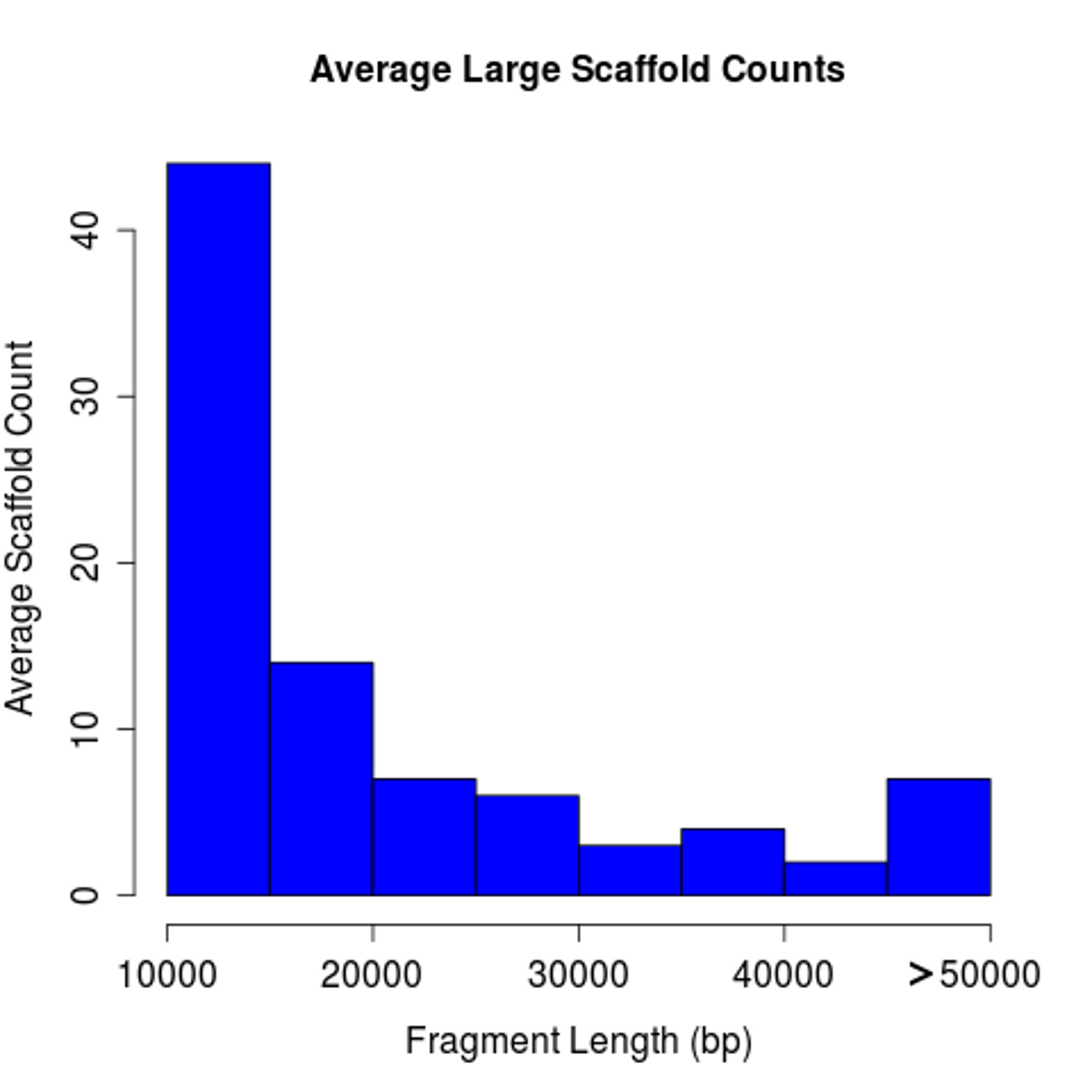

Supplement: Figure S9 — Histogram of Average Large Scaffold Counts in Metagenomic Datasets. This figure shows a histogram of the average frequency of large (>10,000 bp) scaffolds across twenty-five metagenomic datasets collected within Yellowstone National Park. These metagenomic datasets average eighty-seven scaffolds over 10,000 bp, including seven which are over 50,000 bp. (TIF) [file pone.0067337.s009.tif]
